# Examining the Longitudinal Associations between Adjustment Disorder Symptoms and Boredom during COVID-19

**DOI:** 10.3390/bs12090311

**Published:** 2022-08-29

**Authors:** Veerpal Bambrah, Amanda Wyman, Eva Friedman, John D. Eastwood

**Affiliations:** Department of Psychology, Faculty of Health, York University, Toronto, ON M3J 1P3, Canada

**Keywords:** adjustment disorder, preoccupation, failure to adapt, feelings of boredom, trait boredom, longitudinal study, COVID-19

## Abstract

The COVID-19 pandemic has led to a myriad of stressors, underscoring the relevance of adjustment disorder during these extraordinary times. Boredom—as a feeling and as a dispositional characteristic—is an equally pertinent experience during the pandemic that has been cross-sectionally linked to various mental health difficulties. The current longitudinal study expanded on this work, examining the associations between adjustment disorder symptoms and boredom (both as a feeling and as a trait) *over time* during the COVID-19 pandemic. Community participants completed questionnaires three times, rating their trait boredom at Time 1 and their feelings of boredom and adjustment disorder symptoms (*preoccupation* with a pandemic stressor and *failure to adapt*) over the past week at Times 1–3. Latent growth curve analyses found that an increase in feelings of boredom was significantly associated with increased preoccupation with a pandemic stressor and increased difficulties with adapting over time. Additionally, trait boredom significantly predicted changes in preoccupation and the failure to adapt, such that participants high in trait boredom increasingly struggled with these symptoms over time. Our results suggest that increased feelings of boredom and a trait disposition towards boredom can be detrimental to people’s ability to adjust over time to the stressors associated with the pandemic. Boredom, as an aversive state and as a chronic difficulty, may be important to address in treatment approaches for adjustment disorder symptoms during COVID-19.

## 1. Introduction

The coronavirus (COVID-19) outbreak became a global pandemic in March of 2020 [1]. Worldwide, public health measures implemented to control the spread of the disease have led to widespread and extended closures of businesses, schools, workplaces, and other public spaces. Millions have had to adjust to working from home and have faced financial hardship due to dismissals from work or fewer hours of work [2]. Exposure to the threat to health and life, as well as to quarantine, self-isolation, loneliness, job loss, family conflicts, and grief of loved ones, are additional and ongoing stressors associated with the pandemic (e.g., [3,4,5]). Since the first case of COVID-19 was diagnosed in December 2019, the number of known cases and number of deaths are still rising [6], and emerging research indicates an increasing probability of experiencing future pandemics similar to COVID-19 [7]. As such, continued research on the effects of the pandemic on people’s psychological functioning, as well as the factors that mitigate or accentuate such effects, is imperative as it can aid the development of prevention and intervention approaches and more generally facilitate pandemic readiness and resilience.

In light of the above-described stressors associated with the pandemic, adjusting may be problematic for many people. Adjustment disorder is a maladaptive reaction to a stressful event(s) [8]. According to the International Classification of Diseases and Related Health Problems [8], the reaction to the stressful event(s) is characterized by symptoms of preoccupation, such as excessive worry, recurrent and distressing thoughts about the stressor, or constant rumination about the stressor’s implications. Of note, however, recent conceptualizations of preoccupation [9] differentiate it from rumination, outlining that the former consists of time-consuming and factual thoughts that are about the stressor directly (e.g., “I suffer from COVID-19”) and that the latter consists of negative thoughts that may permit one to subtly avoid thinking about the stressor directly (e.g., “This disease and everything related to it is terrible”). In addition to preoccupation, there is a failure to adapt, which is thought to constitute a stress response that manifests in symptoms interfering with everyday functioning, such as concentration or sleep difficulties [8,10]. These symptoms can also be associated with a loss of interest in work, social life, and leisure activities, resulting in significant impairment in occupational, social, and other important areas of functioning (e.g., absenteeism, interpersonal conflict) [8,10].

The COVID-19 pandemic serves as a stressful and large-scale public health, societal, economic, and organizational event that has triggered adjustment disorder in many. Indeed, results from cross-sectional surveys administered between 19 April and 26 May of 2020 to adults in the United States, Canada, Italy, France, China, the “most hard” hit countries, suggest that adjustment disorder is the “emblematic” disorder of COVID-19, finding a higher prevalence of adjustment disorder than of post-traumatic stress disorder (PTSD) in all five countries [11]. Another study found a higher prevalence of increased adjustment disorder symptoms than of depression, generalized anxiety, and PTSD diagnoses among Polish adults surveyed between 25 March and 27 April of 2020 [12]. If left untreated, adjustment disorder can be a “gateway” to other mental health disorders (e.g., major depressive disorder, generalized anxiety disorder, PTSD, substance abuse) [13,14]. As such, it is important to understand adjustment disorder as an outcome in its own right within the context of the pandemic—one that can potentially exacerbate people’s mental health during these extraordinary times. Cross-sectional work examining the risk factors for adjustment disorder during the pandemic suggests that older age, female gender, low income, limited face-to-face contact with others, and exposure to stress/trauma before and during the pandemic are related to more severe symptoms of adjustment disorder [15]. The current study extended upon this work by examining the role of boredom—another pertinent experience within the context of the pandemic—in predicting adjustment disorder symptoms over time during COVID-19.

### 1.1. Boredom and Mental Health during COVID-19

Defined as the aversive feeling of wanting, but being unable, to engage in satisfying activity [16], boredom is not only one of the most frequently experienced feelings during the COVID-19 pandemic (and even the 2003 SARS outbreak), but is also reported to be a key emotional disincentive to complying with social distancing/quarantine measures that are pivotal to containing the transmission of the disease [17,18,19,20,21]. Further, trait boredom—the tendency to experience boredom more frequently and intensely—has been shown to be related to poor adherence to the rules of social distancing [22,23,24,25,26]; but see [27] emerging and conflicting findings related to boredom and risky public health behaviors. 

Associated with low self-control [28,29,30], neuroticism [31], and self-regulatory styles that diminish the pursuit of goals [32,33,34], boredom—as a feeling and as a trait—has been linked to various mental health and behavioral problems prior to the pandemic, such as self-harm, unhealthy eating, depression, anxiety, and addictive behaviors [35,36,37,38]. Cross-sectional studies conducted during the pandemic have specifically found that the feeling of boredom mediates the relationship between loneliness and depression among college students [39]; is one of the most frequently cited reasons for increased alcohol and substance use (e.g., smoking, vaping) among adults in the general population [40,41,42,43,44,45,46]; is inversely related to feelings of happiness [18]; is positively related to symptoms of depression, anxiety, stress, and insomnia among healthy controls and among psychiatric outpatients and inpatients [47,48], as well as a marked fear of COVID-19 among adolescents [49]; and is positively associated with problematic social media use among adult users [50]. Cross-sectional studies examining the link between trait boredom and mental health during COVID-19 have found positive associations between the former and perceived stress among civil servants working from home [51] and among university students [52], as well as depression, fear, obsessions and compulsions, illness anxiety, psychological distress, and internet addiction among adolescents [53] and adults in the general population [54,55,56]. 

Although these previous studies suggest that the feeling of boredom and the tendency to feel bored more frequently and intensely are related to poorer mental health during the pandemic, what is lacking is an understanding of how, if at all, boredom *longitudinally* relates to people’s mental health during COVID-19. The constraints placed on people’s lives during the pandemic have undoubtedly engendered boredom, which, as noted above, is linked to “rule breaking” behaviors that exacerbate the spread of infectious diseases, such as COVID-19. Relatedly, people’s preoccupation and failure to adapt to the stresses associated with the pandemic could worsen if the pandemic persists. Accordingly and importantly, the current longitudinal study sought to explore the longitudinal associations between boredom and adjustment disorder symptoms over time during COVID-19. 

### 1.2. Current Study

Our aim was to examine the link between boredom and adjustment disorder symptoms over time (i.e., across three time points) during the early months of the pandemic. In particular, we hypothesized that (1) the trajectory of the feeling of boredom over time (i.e., between Times 1 and 3) would significantly relate to the trajectory of adjustment disorder symptoms over time, such that those who felt increasingly bored between Times 1 and 3 during the pandemic would increasingly struggle with preoccupation with a pandemic stressor and with the ability to adapt over the same time period. We additionally hypothesized that (2) trait boredom (measured at Time 1) would significantly predict the trajectory of adjustment disorder symptoms over time (i.e., between Times 1 and 3), such that those high in trait boredom would experience increasing preoccupation and increasing difficulties with adapting to a pandemic stressor over time. Our hypotheses were supported. 

## 2. Materials and Methods

### 2.1. Participants and Procedures

**Figure 1:**
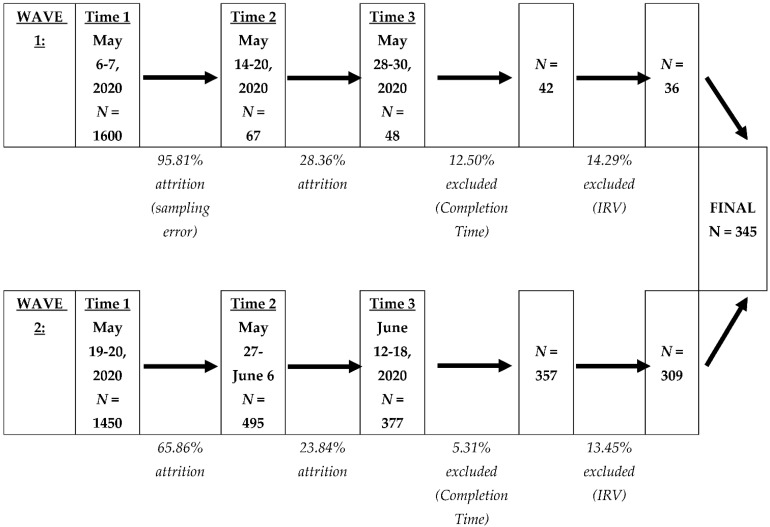
Participant Recruitment, Attrition, and Exclusion. *Note.* Across both waves, the majority of the 345 participants completed Time 3 24 days after they completed questionnaires at Time 1 (*Mode*_days_ between Time 1–3 = 24, *M*_days_ between Time 1–3 = 24.42, *SD*_days_ between Time 1–3 = 1.77). The majority of participants completed questionnaires at Time 2 eight days after they completed questionnaires at Time 1 (*Mode*_days_ between Time 1–2 = 8, *M*_days_ between Time 1–2 = 9.42, *SD*_days_ between Time 1–2 = 2.55). Finally, the majority of participants completed questionnaires at Time 3 16 days after they completed questionnaires at Time 2 (*Mode*_days_ between Time 2–3 = 16, *M*_days_ between Time 2–3 = 15.01, *SD*_days_ between Time 2–3 = 2.74).

A full description of our sample, including how we determined our sample size, has been described elsewhere and is available publicly (https://osf.io/8pexf/, accessed on 31 July 2022). In short, participants from the United States (U.S.) and Canada were recruited via Qualtrics’ Online Panels to complete questionnaires. Participants were contacted three times during the COVID-19 pandemic, specifically between 6 May 2020—approximately two months after the U.S. declared the COVID-19 pandemic a national emergency—and 18 June 2020. In light of a sampling error that prevented us from contacting all of the participants who had completed Time 1 to complete Time 2, we recruited participants in two waves (Wave 1 and Wave 2). The number of participants at the three data collection time points for Waves 1 and 2, as well as dates of contact, participant attrition, and participant exclusion, are indicated in Figure 1. We determined whether a participant was a valid responder based on (a) the time it took to complete questionnaires and (b) insufficient effort in responding. For (a), a participant’s data was excluded across all three time points if they completed all questionnaires in an unrealistically short time (defined as completing Time 1 in under 400 s or Time 2 or Time 3 in under 300 s) or in an unrealistically long time (defined as completing Times 1, 2, or 3 in over 36,000 s). For (b), we calculated an intra-individual response variability (IRV) value, which is an index of psychological disengagement in the form of providing similar responses across positively- and negatively-worded items [57], for each participant at all three time points on two questionnaires that possess both positively- and negatively-worded items. Participants with an IRV value greater than 2 standard deviations below the mean IRV on any one of the two questionnaires at any time point were excluded across all three time points. The final sample at Time 3 across Waves 1 and 2 consisted of 345 participants (*M*_age_ = 49.26, *SD*_age_ = 16.68, range_age_ = 18–88, 234 females, 110 males, 1 did not disclose their gender, 0 indicated ‘other’ gender), with the majority residing in the U.S. (Wave 1 = 100.00%, Wave 2 = 99.68%). At the time of the study, the majority of participants were living with someone else (*N* = 252, 73.04%; 1 participant did not report their living arrangement), were not enrolled in school (*N* = 311, 90.14%), were unemployed (*N* = 219, 63.48%), and had a yearly household income of $50,000 or less (*N* = 207, 60.00%). 

### 2.2. Questionnaires

Participants completed a large number of questionnaires of which a subset of responses are reported, described, and analyzed here. The full set of questionnaires can be accessed at https://osf.io/8pexf/, accessed on 31 July 2022. Table 1 presents the coefficient omega estimates and descriptive statistics (range, mean, standard deviation) of all of the measures that were administered at Times 1, 2, and 3 of the current study.

**Trait Boredom.** At Time 1, participants completed the Trait Boredom Scale (TBS), a newly developed six-item measure of an individual’s experience of boredom—i.e., the tendency to often feel bored because of diminished agency [58]. Participants indicated the extent to which each item (e.g., “I often do not know what I want to do”) applies to them and their life in general using a 7-point scale (1 = *strongly disagree* to 7 = *strongly agree*), with a higher total score indicating greater trait boredom. Confirmatory factor analyses (CFA) conducted with community and undergraduate research participants from other studies suggest that a one-factor structure of the six-item TBS fits the data well and possesses good-to-excellent internal reliability: Comparative Fit Index (CFI) range = 0.977 to 0.998, Root Mean Square Error of Approximation (RMSEA) range = 0.025 to 0.090, Standardized Root Mean Square Residual (SRMR) range = 0.014 to 0.029, and ω range = 0.84 to 0.93 [58]. A CFA using the maximum likelihood with robust standard errors (MLR) estimation method found that a one-factor structure of the six-item TBS had an acceptable-to-good fit with the data of the current study (at Time 1): CFI = 0.969, RMSEA = 0.105, SRMR = 0.033. The internal reliability for the TBS was excellent (see Table 1).

**Pre-Pandemic Stress.** At Time 1, participants responded to one question about the life stressors that they had experienced prior to the COVID-19 pandemic. Adapted from the Adjustment Disorder-New Module 20 Questionnaire (ADNM-20) [59], participants were provided a list of 16 stressful life events (e.g., divorce/separation, death of a loved one, unemployment, assault) and they indicated which event(s), if any, happened to them during the 2 years before the pandemic that remains a “very strong” burden to them/has burdened them in the past six months. Participants were provided space to note stressful experiences that were not included on the list. A higher total score indicated a greater number of burdensome pre-pandemic stressors. 

**Boredom.** At Times 1, 2, and 3, participants completed the short-form Multidimensional State Boredom Scale (MSBS), which measured their boredom over the past week at each time point. Participants rated six items (e.g., “I felt like I was sitting around waiting for something to happen”) from the 28-item MSBS [16] using a 7-point scale (1 = *strongly disagree* to 7 = *strongly agree*), with a higher total score indicating greater boredom over the past week. A series of CFAs conducted with community and undergraduate research participants from other studies indicate that a one-factor structure of the six-item MSBS fits the data well and possesses good internal reliability: CFI range = 0.956 to 0.991, RMSEA range = 0.048 to 0.086, SRMR range = 0.024 to 0.045, and ω range = 0.79 to 0.85 (e.g., [60]). A CFA confirmed that a one-factor structure of the six-item MSBS had an acceptable-to-good fit with the data of the current study across all three time points: CFI range = 0.961 to 0.981, RMSEA range = 0.079 to 0.107, and SRMR range = 0.028 to 0.042. The internal reliability of the six-item MSBS across all three time points was good (see Table 1).

**Adjustment Disorder.** At Times 1, 2, and 3, participants completed the brief Adjustment Disorder-New Module 8 (ADNM-8) [61], which measured two core symptoms of adjustment disorder, specifically preoccupation with a stressor(s) and failure to adapt to the stressor(s). In the current study, participants were asked to think back to any stressful event(s) that they had experienced during the COVID-19 pandemic and answer questions about their reactions to the stressful event(s) over the past week. Participants rated eight items using a 4-point scale (1 = *never* to 4 = *often*), which were summed to create a total score, as well as subscale scores: preoccupation (e.g., “I had to think about the stressful situation repeatedly”) and failure to adapt (e.g., “Since the stressful situation, I did not like working or carrying out the necessary tasks in everyday life”). The total score ranges from 8 to 32 and a score of 18.5 or higher is indicative of possible Adjustment Disorder. The internal consistency of the total scale across time points was excellent, as well as good-to-excellent across time points for the four-item preoccupation subscale and the three-item failure to adapt subscale (see Table 1).

### 2.3. Data Analytic Plan

Data were analyzed using R (version 4.2.1) [62], with the packages *reshape2* (version 1.4.4) [63] and *lavaan* (version 0.6–12) [64]. 

Using the R package *lavaan*, latent growth curve models (LGCMs) were estimated to examine the linear change in participants’ preoccupation and failure to adapt over the course of the study (i.e., between Times 1 and 3—an average of 24 days between 6 May 2020 and 18 June 2020). Two latent variables were estimated for each dependent variable. The intercept latent variable was created by setting the paths to the dependent variable of interest at each time point to 1, which indicates the average score of the dependent variable at Time 1. The slope latent variable was created by setting the paths to the dependent variable of interest at each time point to 0, 1, and 2, respectively. A positive slope coefficient would indicate a linear increase in the dependent variable over time; a negative slope coefficient would indicate a linear decrease in the dependent variable over time. Multiple regression models were then estimated to assess whether predictor variables are significantly related to the intercepts (i.e., initial levels) and slopes (i.e., changes over time) of the dependent variables, the preoccupation and failure to adapt subscales of the ADNM-8. The predictor variables were the intercept of boredom and the slope of boredom (felt over the past week) across time points, as well as trait boredom (at Time 1). Age, gender (1 = *male*, 2 = *female*), income (1 = *$50,000 or less*, 2 = *$50,001 or more*), and living arrangement (1 = *not living with someone*, 2 = *living with someone*) were included in each model as prior work has shown these to have significant impacts on people’s adjustment disorder symptoms and psychological distress during the pandemic (e.g., [15,65]). Pre-pandemic stress was also included in each model as any changes in participants’ adjustment disorder symptoms during the pandemic may be influenced by the number of burdensome stressors they experienced prior to the pandemic. Of particular interest were whether (1) initial levels (i.e., intercept) of boredom and trait boredom significantly predict initial levels of the dependent variable; and (2) the trajectory (i.e., slope) of boredom and trait boredom significantly predict changes over time in the dependent variable.

## 3. Results

### 3.1. Descriptive Analyses 

A series of Independent *t*-tests found no statistically significant differences between participants who were recruited in Wave 1 (*n* = 36) and participants who were recruited in Wave 2 (*n* = 309) on the TBS total score at Time 1; the short-form MSBS total score across all three time points; the ADNM-8 total and subscale scores across all three time points; age; and the number of pre-pandemic stressors reported (all *p*’s > 0.05). Additionally, Chi-square tests indicated no statistically significant differences between groups on gender (i.e., 1 = *male*, 2 = *female*; the participant who did not report their gender was excluded from data analyses); income (i.e., 1 = *$50,000 or less*, 2 = *$50,001 or more*); and living arrangement (i.e., 1 = *not living with someone*, 2 = *living with someone*; the participant who did not report their living arrangement was excluded from data analyses) (all *p*’s > 0.05). Accordingly, all 343 participants were analyzed together. 

In the current study, the largest proportion of participants (*N* = 115, 33.33%) reported at least one life stressor that preceded the COVID-19 pandemic and has burdened them (range = 0–10). A full description of the type of burdensome stressors experienced by our sample prior to the pandemic is reported elsewhere (https://osf.io/8pexf/, accessed on 31 July 2022).

Table 1 shows descriptive statistics of all continuous variables in the study. The average total score on the ADNM-8 at Times 1, 2, and 3 was 18.15, 17.99, 17.31, respectively, which suggests sub-clinical pandemic-related adjustment difficulties over the course of the study. Using the above-noted cut-off for the ADNM-8, nearly half of participants (*N* = 165, 47.83%) met criteria for possible Adjustment Disorder at Times 1 and 2, and 44.06% of participants (*N* = 152) met criteria for possible Adjustment Disorder at Time 3. 

Using the R package *reshape2*, spaghetti plots were created with a random subsample of the data (*n* = 50) to examine whether a linear growth model would be appropriate for the data. The two plots did not indicate deviations from a linear growth in participants’ preoccupation and failure to adapt and we did not observe any curvilinear patterns of change in preoccupation and failure to adapt between Times 1 and 3. As such, we proceeded to estimate linear growth models to examine the linear changes over time in these outcomes and the predictors of such changes.

Table 2 summarizes the results. There were significant linear decreases between Time 1 and Time 3 in participants’ preoccupation with a stressor(s) that they had experienced during the pandemic (*B* = −0.20, *SE* = 0.07, *p* = 0.005, 95% CI = [−0.34, −0.06], *β* = −0.20) and in their difficulties with adapting to such stressor(s) (*B* = −0.18, *SE* = 0.05, *p* = 0.001, 95% CI = [−0.29, −0.07], *β* = −0.31). Subsequently, we explored the predictors of the intercept latent variable and slope latent variable for both ADNM-8 subscales. 

### 3.2. Predictors of Initial and Changes in Preoccupation

Initial levels of boredom (*B* = 0.17, *SE* = 0.02, *p* < 0.001, 95% CI = [0.13, 0.21], *β* = 0.47) and trait boredom (*B* = 0.08, *SE* = 0.02, *p* < 0.001, 95% CI = [0.04, 0.11], *β* = 0.25) were significant and unique positive predictors of participants’ initial levels of preoccupation, over and above each other and all covariates (i.e., age, gender, income, living arrangement, and pre-pandemic stress). Participants with a greater number of pre-pandemic stressors struggled with higher preoccupation at Time 1 (*B* = 0.35, *SE* = 0.08, *p* < 0.001, 95% CI = [0.20, 0.51], *β* = 0.22). Participants’ age, gender, income, and living arrangement were non-significant predictors of their preoccupation at Time 1. 

The slope of boredom (*B* = 0.83, *SE* = 0.35, *p* = 0.017, 95% CI = [0.15, 1.51], *β* = 0.82) was significantly and positively associated with the change over time in participants’ preoccupation, over and above age, gender, income, living arrangement, pre-pandemic stress, and trait boredom. In other words, participants’ preoccupation with a stressor that they experienced during the pandemic significantly increased over time as their feelings of boredom increased, while controlling for these other participant characteristics. Trait boredom (*B* = −0.03, *SE* = 0.01, *p* = 0.001, 95% CI = [−0.04, −0.01], *β* = −0.24) and pre-pandemic stress (*B* = −0.09, *SE* = 0.04, *p* = 0.024, 95% CI = [−0.17, −0.01], *β* = −0.16) also significantly and uniquely predicted the change over time in participants’ preoccupation with a pandemic stressor, over and above all other variables in the model. 

We examined how the slope of preoccupation with a pandemic stressor changed for participants with different levels of trait boredom and pre-pandemic stress, specifically 2 standard deviations above the mean TBS score and pre-pandemic stress score, 2 standard deviations below the mean TBS score and pre-pandemic stress score, and at the mean score of these variables. See the Supplementary Materials for the executed R code and corresponding slope values. Among participants with a TBS score 2 standard deviations above the mean score, the slope of preoccupation was positive, indicating that participants higher in trait boredom were *increasingly* preoccupied over time with a stressor they had experienced during the pandemic. Among participants with a TBS score 2 standard deviations below the mean score, the slope of preoccupation was negative, suggesting that participants lower in trait boredom were *decreasingly* preoccupied with the stressor over time. A similar pattern emerged for pre-pandemic stress, such that those burdened by a greater number of stressors from before the pandemic increasingly struggled with preoccupation over time, whereas those burdened by a fewer number of pre-pandemic stressors decreasingly struggled with preoccupation over time. 

### 3.3. Predictors of Initial and Changes in the Failure to Adapt

Additionally, initial levels of boredom (*B* = 0.17, *SE* = 0.01, *p* < 0.001, 95% CI = [0.14, 0.20], *β* = 0.62) and trait boredom (*B* = 0.05, *SE* = 0.01, *p* < 0.001, 95% CI = [0.02, 0.07], *β* = 0.19) were significant and unique positive predictors of participants’ failure to adapt to a pandemic stressor at the start of the study, over and above each other and all covariates (i.e., age, gender, income level, living arrangement, and pre-pandemic stress). Participants with a greater number of pre-pandemic stressors (*B* = 0.17, *SE* = 0.06, *p* = 0.005, 95% CI = [0.05, 0.29], *β* = 0.14), as well as participants with a yearly household income of $50,000 or less (*B* = −0.49, *SE* = 0.22, *p* = 0.027, 95% CI = [−0.93, −0.06], *β* = −0.11), struggled more to adapt to a pandemic stressor at Time 1. Age, gender, and living arrangement were non-significant predictors of the failure to adapt at Time 1.

The slope of boredom (*B* = 0.51, *SE* = 0.22, *p* = 0.023, 95% CI = [0.07, 0.94], *β* = 0.86) was significantly and positively related to the change over time in participants’ failure to adapt to a pandemic stressor, over and above all other variables in the model. Put differently, participants’ difficulties with adapting to a stressor that they had experienced during the pandemic significantly increased over time as their feelings of boredom increased. Trait boredom (*B* = −0.02, *SE* = 0.01, *p* = 0.011, 95% CI = [−0.03, −0.00], *β* = −0.25) also significantly and uniquely predicted the change over time in difficulties with adapting, over and above all other variables. 

Participants with a TBS score 2 standard deviations above the mean score *increasingly* struggled to adapt over the course of the study (as evidenced by a positive slope value), whereas participants with a TBS score 2 standard deviations below the mean score *improved* in their ability to adapt (as evidenced by a negative slope value). See the Supplementary Materials. 

## 4. Discussion

Adjustment disorder and boredom are prevalent during the COVID-19 pandemic. To the very best of our knowledge, no other study has examined the longitudinal relationship between boredom—as a dynamic affective experience and as a trait disposition—and mental health symptoms during the pandemic. The current study builds on pre-existing cross-sectional literature, using a longitudinal design to advance our understanding of the link between boredom and mental health *over time* during the pandemic, with a specific focus on adjustment disorder symptoms. 

Overall, participants’ preoccupation with a stressor that they had experienced during the pandemic and their difficulties with adapting to the stressor significantly decreased over time during the pandemic (i.e., between 6 May and 18 June 2020). These findings coincide with other longitudinal research that explored the trajectory of psychological functioning among the general U.S. population in the early months of the COVID-19 pandemic. For example, one longitudinal study found that there was a significant increase among American adults in psychological distress (e.g., feeling depressed, feeling worried) between March and April 2020, but a return to pre-pandemic levels by June 2020 [66]. Relatedly, another longitudinal study found that past-month prevalence of serious psychological distress in May 2020 was just as high as past-year prevalence of serious psychological distress in February 2019 [67]. Another longitudinal study analyzing real-time suicide data from official government sources found that observed suicide rates in the U.S. from 1 April to 30 July 2020 were significantly lower than model expectations during this timeframe, and that this pattern of results remained largely unchanged when including data up to 31 October 2020 [68]. Finally, longitudinal evidence on loneliness and social connection found that, among Americans surveyed in January, March, and April 2020, there were no mean level-changes in loneliness but rather a significant increase in perceptions of social support [69], which suggests that people found other ways to adjust and fulfill their fundamental need to socially connect when familiar channels were blocked. Overall, our findings dovetail with this research, suggesting that people’s adjustment related to the pandemic did not deteriorate over the course of the early months of the pandemic but rather improved. Notably, the current study further built on this research by demonstrating that people’s feelings of boredom and trait boredom significantly influence the trajectory of their adjustment disorder symptoms over time during COVID-19. 

While controlling for people’s age, gender, income, living arrangement, and pre-pandemic stress, we found that at beginning of the study (at Time 1), feeling more bored over the past week and higher trait boredom significantly and uniquely predicted greater difficulties with preoccupation and with adapting to a pandemic stressor. These results coincide with the early reviewed literature on the cross-sectional relationships of the feeling of boredom and trait boredom with various mental health outcomes during the pandemic. Most notably, over the course of the study (between Time 1 and Time 3), and over and above all other participant characteristics, increased feelings of boredom was significantly and uniquely associated with increased preoccupation and increased difficulties with adapting over the same time period. Moreover, higher trait boredom significantly and uniquely predicted increased preoccupation and increased difficulties with adapting. In keeping with the above-reviewed conceptualizations of these adjustment disorder symptoms, the results of the current study suggest that people whose feelings of boredom increased during the pandemic, as well as people who generally tend to feel bored more frequently and intensely, increasingly struggled with time-consuming, factual, and distressing thoughts about a stressful event they had experienced during the pandemic and they increasingly struggled with concentration, sleep, working, and carrying out other necessary tasks in everyday life.

There are key defining characteristics of the feeling of boredom and of trait boredom that may account for why they were significantly and positively linked to participants’ initial and increased preoccupation and failure to adapt over time during the pandemic. The feeling of boredom can be usefully defined as the aversive emotional concomitant of “unused cognitive potential” [70], engendered by suboptimal levels of challenge, constraint, monotony, and devalued activities [71]. Additionally and notably, boredom is rooted in a “desire bind” [72], which means that when a person feels bored, they cannot find anything they want to do in their current environment, but they desperately want to do something. Emerging work [60] also suggests that experimentally manipulating feelings of boredom causes a significant increase in *self-directed attention*, such that a person is more focused on their thoughts and feelings after they have been made to feel bored. Relatedly, several studies also show that trait boredom is positively linked to trait-based measures of self-directed attention and negatively linked to trait-based measures of *self-knowledge*, which suggests that people prone to frequent and intense boredom tend to focus on their thoughts and feelings but tend to lack a clear understanding of these experiences (see [60] for a review)—and thereby, are alienated from their desires and goals. In the context of the pandemic, people were faced with various stressors, along with widespread and extended closures and physical distancing measures that left many without access to cognitively engaging, challenging, and meaningful resources, such as social supports, places of worship, and recreational facilities. Speaking to the above-described characteristics of boredom (as a state and trait), it is plausible that those who increasingly felt bored over time and those who are more prone to boredom directed more attention to their thoughts and feelings but struggled to understand these experiences—unable to articulate and pursue actionable desires and goals—and thus, were increasingly preoccupied with time-consuming and distressing thoughts about a pandemic stressor and increasingly unable to concentrate and carry out day-to-day tasks in spite of the stressor. Future research could examine if the relations of feeling bored and trait boredom with these adjustment disorder symptoms over time during the pandemic involve or are independent of self-directed attention and self-knowledge. For example, it could be that self-directed attention mediates the relationship between increased feelings of boredom and increased preoccupation and difficulties with adapting. It could also be that people high in trait boredom struggle more with increasing adjustment disorder symptoms if they lack self-knowledge (i.e., trait self-knowledge might moderate the relationship between trait boredom and increasing adjustment disorder symptoms). 

That trait boredom positively predicted initial and increased preoccupation and difficulties with adapting underscores the “self-regulatory profile” of the boredom-prone person. Prior research has shown that trait boredom is associated with certain trait modes of self-regulation that diminish the pursuit of one’s goals. More specifically, early work [32] suggests that trait boredom is linked to state orientation, a change-preventing mode of self-regulation, specifically finding that trait boredom is positively related to both state-oriented “preoccupation” and “hesitation”—the former characterized by persevering and intrusive thoughts associated with unpleasant experiences and the latter characterized by an impaired initiation of an intended action. Two more recent studies [33,34] have found that trait boredom is positively associated with an “Assessment” mode of self-regulation, which is characterized by an exhaustive and ruminative evaluative orientation (i.e., making sure to “do the right thing”) that stifles or delays the initiation of goal-pursuit, but is negatively associated with a “Locomotion” mode of self-regulation, which emphasizes implementing action and is characterized by moving from one goal to another (i.e., “just do it”). Most recently, one study [73] found that trait boredom is negatively associated with metacognitive temporal control, which is the ability to manage one’s temporal focus so that one’s thoughts are focused on the here-and-now (as opposed to on unwanted and intrusive thoughts related to the past or future). Situating this pre-existing research in the context of the current study might suggest that the boredom-prone person experiences increasing difficulties with preoccupation and with adapting to pandemic stressors because of their tendency towards state-oriented “preoccupation” and “hesitation” and an “Assessment” evaluative orientation, as well as their limited ability to focus their thoughts on the present. Future research that closely examines the mediating roles of these facets of self-regulation in the longitudinal relationships between trait boredom and adjustment disorder symptoms may provide us with a more nuanced understanding of *how* those who struggle with frequent and intense boredom experience increasing difficulties with preoccupation and with adapting to pandemic stressors. 

### Implications and Future Directions

The current study is the first to illustrate that the feeling of boredom and trait boredom are significantly and uniquely linked to increasing mental health symptoms during the pandemic, specifically increasing symptoms of adjustment disorder. This work not only furthers our understanding of boredom in the context of mental health outcomes during the pandemic (i.e., what outcomes boredom is related to and in what manner), but also may inform potential prevention and intervention efforts during the pandemic that seek to ameliorate people’s adjustment disorder symptoms. Broadly, the current study highlights who may particularly benefit from treatments for adjustment disorder (i.e., those who chronically struggle with boredom), as well as areas of focus for treatment (i.e., learning to adaptively cope with and respond to the aversive feeling of boredom). Of course, however, more research is needed to understand exactly how increased feelings of boredom and trait boredom are associated with increased symptoms of adjustment disorder during the pandemic; a more nuanced understanding of the underlying mechanisms (e.g., the roles of self-directed attention, self-knowledge, and/or self-regulatory modes) in turn can help refine the focus of treatments for adjustment disorder.

The current study had many strengths, such as a large sample size from across the U.S., a longitudinal design, and multiple time points of assessment, including one within the first 2 months of the U.S.’s national emergency response. However, there were some limitations. First, we conducted our study with a community sample and we did not assess pre-pandemic psychopathology. Although our work has general implications for understanding and treating adjustment disorder symptoms during the pandemic, we cannot extrapolate these findings to clinical populations and we could not account for the potential impact of pre-existing psychopathology. Second, our study collected self-report data online, as well as asked participants to retrospectively recall pre-pandemic experiences (e.g., stressors) after the pandemic began. These methods may engender response and recall biases; however, in order to collect the most accurate data possible, we implemented the use of well-validated measures and thoroughly reviewed our sample for punctual and reliable responders. Third, because participants’ feelings of boredom and adjustment disorder symptoms were measured at each of the three time points, we do not have temporal precedence and thus, cannot make conclusions about the cause-effect relationship between these variables—i.e., that increased boredom precedes increased adjustment disorder symptoms. Fourth, additional longer-term longitudinal studies are needed to understand the nature of the relationships between feelings of boredom, trait boredom, and adjustment disorder symptoms beyond the time points in this study. These studies will be especially critical when examining long-term and residual effects of the pandemic. Relatedly, if adjustment disorder symptoms do exacerbate and lead to other mental health difficulties for some people, it will be important to understand how boredom plays a role in this change.

Importantly, the novel understanding of the longitudinal relationship between boredom and symptoms of adjustment disorder introduces the opportunity to ask additional questions about the nature of this relationship. Firstly, our models controlled for age, gender, income, living arrangement, and pre-pandemic stress, thus emphasizing that when these factors are held constant, people’s increased feelings of boredom and trait boredom uniquely predict increases in their preoccupation with a pandemic stressor and difficulties adapting to the stressor. Future research could explore the moderating role of these factors, thus elucidating if and how age, gender, income, living arrangement, and pre-pandemic stress enhance or diminish the impact of boredom on people’s adjustment disorder symptoms over time. Second, future research that shows that increases in people’s boredom precede increases in their preoccupation and difficulties adapting would not only clarify the directional relationship between these pandemic-relevant experiences, but, relatedly, would set the stage for exploring how characteristics of boredom (e.g., self-directed attention) influence people’s adjustment difficulties. Third, in addition to boredom, the current study introduces the possibility that other related and pandemic-relevant experiences may influence the trajectory of adjustment disorder symptoms during COVID-19. For example, adverse life events, such as those associated with the pandemic, can unsettle one’s life meaning and purpose, which may otherwise help people cope and react adaptively in the midst of stressors [74,75]. Notably, experimental work shows that manipulating perceptions of life meaning significantly changes boredom, such that people with low meaning feel significantly more bored than those with high meaning, and longitudinal work shows that life meaning predicts lower boredom across time [76]. As prior research has showed, boredom is differentiated from anger, frustration, and sadness insofar as it involves a perception that one’s situation is meaningless (e.g., [77,78,79]). Thus, future research might consider examining how, if at all, disrupted meaning predicts people’s adjustment disorder symptoms during the pandemic—i.e., either in place of or as a driver of feelings of boredom.

## 5. Conclusions

To conclude, our longitudinal study extended on prior cross-sectional research by examining how boredom and adjustment disorder symptoms—both pertinent and important experiences during COVID-19—are related to each other *over time* during the pandemic. We found that boredom negatively impacts people’s adjustment to a pandemic stressor(s) over time during COVID-19. Our results suggest that increased feelings of boredom and trait boredom are linked to increases in people’s preoccupation with a pandemic stressor over time, as well as increased difficulties with adapting to the stressor. Those who experience exacerbating feelings of boredom and who are generally prone to frequent and intense boredom may be particularly vulnerable to worsening adjustment disorder symptoms. Prevention and intervention approaches that address people’s adjustment difficulties during the pandemic should consider targeting those who struggle with chronic boredom, as well as consider focusing treatment on how to effectively respond to the aversive feeling of being cognitively unengaged during these extraordinary times.

## Figures and Tables

**Table 1:** Coefficient Omega Estimates, Ranges, Means, and Standard Deviations for Continuous Variables at Times 1, 2, and 3.

Variable		Time 1			Time 2			Time 3		
	Range	ω	*M*	*SD*	ω	*M*	*SD*	ω	*M*	*SD*
*Trait Boredom*	6.00–42.00	0.90	22.86	9.08	-	-	-	-	-	-
*Boredom*	6.00–42.00	0.87	21.74	9.13	0.89	21.68	9.51	0.89	21.29	9.56
*ADNM-8 Total*	8.00–32.00	0.93	18.15	6.50	0.95	17.99	6.99	0.94	17.31	6.91
*Preoccupation*	4.00–16.00	0.90	9.21	3.39	0.93	9.09	3.63	0.92	8.80	3.58
*Failure to Adapt*	3.00–12.00	0.82	6.78	2.67	0.87	6.68	2.80	0.85	6.41	2.77

**Table 2:** Results of Latent Growth Curve Models.

Dependent Variable *Predictor*	CFI	RMSEA	SRMR	Intercept *B* (SE)	95% CI	Slope *B* (SE)	95% CI	Intercept Variance	Slope Variance
**Preoccupation**	**1.00**	**0.00**	**0.01**	9.22 (0.18) ***	**8.86, 9.57**	−0.20 (0.07) **	**−0.34, −0.06**	10.20 (1.08) ***	1.02 (0.38) **
*Feeling Bored ^a^*				0.17 (0.02) ***	0.13, 0.21	0.83 (0.35) *	0.15, 1.51	-	-
*Trait Boredom*				0.08 (0.02) ***	0.04, 0.11	−0.03 (0.01) **	−0.04, −0.01	-	-
*Pre-Pandemic Stress*				0.35 (0.08) ***	0.20, 0.51	−0.09 (0.04) *	−0.17, −0.01	-	-
*Age*				−0.02 (0.01)	−0.04, 0.00	0.00 (0.00)	−0.01, 0.01	-	-
*Gender*				0.17 (0.30)	−0.42, 0.76	0.02 (0.15)	−0.27, 0.32	-	-
*Income*				−0.27 (0.29)	−0.85, 0.31	0.23 (0.15)	−0.05, 0.52	-	-
*Living Arrangement*				−0.41 (0.33)	−1.06, 0.25	−0.14 (0.17)	−0.46, 0.19	-	-
**Failure to Adapt**	**1.00**	**0.00**	**0.01**	6.80 (0.14) ***	**6.52, 7.08**	−0.18 (0.05) **	**−0.29, −0.07**	5.87 (0.65) ***	**0.35 (0.24)**
*Feeling Bored ^a^*				0.17 (0.01) ***	0.14, 0.20	0.51 (0.22) *	0.07, 0.94	-	-
*Trait Boredom*				0.05 (0.01) ***	0.02, 0.07	−0.02 (0.01) *	−0.03, −0.00	-	-
*Pre-Pandemic Stress*				0.17 (0.06) **	0.05, 0.29	−0.06 (0.03)	−0.12, 0.00	-	-
*Age*				−0.01 (0.01)	−0.03, 0.00	−0.00 (0.00)	−0.01, 0.01	-	-
*Gender*				0.44 (0.23)	−0.01, 0.89	−0.15 (0.11)	−0.38, 0.07	-	-
*Income*				−0.49 (0.22) *	−0.93, −0.06	0.16 (0.11)	−0.05, 0.38	-	-
*Living Arrangement*				−0.14 (0.25)	−0.64, 0.35	−0.15 (0.13)	−0.40, 0.09	-	-

*Note.* ^a^ The intercept of feeling bored was used to predict the intercept of the dependent variable and the slope of feeling bored was used to predict the slope of the dependent variable. * *p* < 0.05, ** *p* < 0.01, *** *p* < 0.001.

## Data Availability

Subject to the ethical requirements of the York University Human Participants Research Committee and the Canadian Tri-Council policy statement on ethical conduct for research involving humans, all data, analysis code, and research materials underlying this study will be made available to researchers upon submission of an approved ethics protocol from their academic or research institution (or equivalent) to the corresponding author. Interested researchers can submit queries related to data access to the corresponding author.

## Supplementary Materials

The following supporting information can be downloaded at: https://www.mdpi.com/article/10.3390/bs12090311/s1.
