# Supplementary material for: Examining the Longitudinal Associations between Adjustment Disorder Symptoms and Boredom during COVID-19
Source: Behav Sci (Basel). 2022 Aug 29;12(9):311. doi: 10.3390/bs12090311 (PMC9495664; doi:10.3390/bs12090311)
Supplement: Supplementary file 1 [file behavsci-12-00311-s001.zip › behavsci-1869774-supplementary.pdf]

# Supplementary Materials:
# Examining the Longitudinal Associations between Adjustment Disorder Symptoms and Boredom during COVID-19

**R code and Corresponding Slope Values of Dependent Variables (based on Different Levels of TBS and Pre-pandemic Stress)**

**Preoccupation with a pandemic stressor:**
*Slope of preoccupation among participants 2 SD above the mean TBS score:*
(slope_plus2sd <- 0.61 +
-0.03*(mean(datadnmfull$TBS_6_T1)) +
2*sd(datadnmfull$TBS_6_T1)) = **18.09**

*Slope of preoccupation among participants 2 SD below the mean TBS score:*
(slope_minus2sd <- 0.61 +
-0.03*(mean(datadnmfull$TBS_6_T1)) –
2*sd(datadnmfull$TBS_6_T1)) = **-18.24**

*Slope of preoccupation among participants at the mean TBS score:*
(slope_mean <- 0.61 +
-0.03*(mean(datadnmfull$TBS_6_T1))) = **-0.08**

*Slope of preoccupation among participants 2 SD above the mean Pre-pandemic stress score:*
(slope_plus2sd <- 0.61 +
-0.09*(mean(datadnmfull$PRECOVIDSTRESSORS)) +
2*sd(datadnmfull$PRECOVIDSTRESSORS)) = **3.94**

*Slope of preoccupation among participants 2 SD below the mean Pre-pandemic stress score:*
(slope_minus2sd <- 0.61 +
-0.09*(mean(datadnmfull$PRECOVIDSTRESSORS)) –
2*sd(datadnmfull$PRECOVIDSTRESSORS)) = **-3.13**

*Slope of preoccupation among participants at the mean Pre-pandemic stress score:*
(slope_mean <- 0.61 +
-0.09*(mean(datadnmfull$PRECOVIDSTRESSORS))) = **0.40**

**Failure to Adapt:**
*Slope of failure to adapt among participants 2 SD above the mean TBS score:*
(slope_plus2sd <- 0.78 +
-0.02*(mean(datadnmfull$TBS_6_T1)) +
2*sd(datadnmfull$TBS_6_T1)) = **18.49**

*Slope of failure to adapt among participants 2 SD below the mean TBS score:*
(slope_minus2sd <- 0.78 +
-0.02*(mean(datadnmfull$TBS_6_T1)) –
2*sd(datadnmfull$TBS_6_T1)) = **-17.84**

*Slope of failure to adapt among participants at the mean TBS score:*
(slope_mean <- 0.78 +
-0.02*(mean(datadnmfull$TBS_6_T1))) = **0.32**
